# Potential International Spread of Multidrug-Resistant Invasive *Salmonella enterica* Serovar Enteritidis

**DOI:** 10.3201/eid1807.120063

**Published:** 2012-07

**Authors:** Irene Rodríguez, M. Rosario Rodicio, Beatriz Guerra, Katie L. Hopkins

**Affiliations:** Federal Institute for Risk Assessment, Berlin, Germany (I. Rodríguez, B. Guerra);; Universidad de Oviedo, Oviedo, Spain (M.R. Rodicio);; and Health Protection Agency, London, UK (K.L. Hopkins)

**Keywords:** hybrid plasmid, antimicrobial resistance, virulence, PFGE, MLST, *Salmonella enterica* serovar Enteritidis, Africa, salmonellosis, salmonellae

## Abstract

In developing countries, *Salmonella enterica* serovar Enteritidis causes substantial illness and death, and drug resistance is increasing. Isolates from the United Kingdom containing virulence-resistance plasmids were characterized. They mainly caused invasive infections in adults linked to Africa. The common features in isolates from these continents indicate the role of human travel in their spread.

Worldwide, nontyphoidal *Salmonella enterica* is a major cause of foodborne illness, and Enteritidis is one of the most commonly reported serovars (www.who.int/salmsurv/links/GSSProgressReport2005.pdf). In industrialized countries, *S*. *enterica* serovar Enteritidis commonly causes self-limiting gastroenteritis, for which treatment with antimicrobial drugs is usually not needed. However, in developing countries, this serovar, together with serovar Typhimurium, frequently causes invasive infections and substantial illness and death among young children with underlying diseases and among adults with HIV infection (*1*). Although antimicrobial drug resistance is not as high in *S. enterica* serovar Enteritidis as in other zoonotic disease serovars, multidrug-resistance (resistance to >4 antimicrobial drugs) has been increasingly reported (*2*), threatening treatment success for patients with severe infections. In recent years, in association with multidrug resistance, another trend has arisen: the emergence of virulence-resistance (VR) plasmids; these are hybrid plasmids that harbor resistance (R) and virulence (V) determinants. The appearance of these plasmids is of concern because they could lead to the co-selection of virulence (in addition to resistance) through the use of antimicrobial drugs (*3**,**4*). One such plasmid, pUO-SeVR1, has been recently reported in a multidrug-resistant (MDR) clinical isolate of *S. enterica* serovar Enteritidis (CNM4839/03) from Spain (*5*). This mobilizable plasmid of ≈100 kb derives from pSEV, the serovar-specific V plasmid of *S. enterica* serovar Enteritidis, and carries most of its V determinants, including the *spvRABCD* locus (*Salmonella* plasmid virulence). This plasmid greatly increases the ability of salmonellae to proliferate intracellularly and has been associated with severe infections in humans (*6*). The plasmid also harbors several R genes—*bla*_TEM-1,_ *catA2, strA*-*strB, sul1, sul2, tet*(A)—and a class-1 integron with the 700-bp/*dfrA7* variable region, which confer resistance to ampicillin, chloramphenicol, streptomycin, sulfonamides, tetracycline, and trimethoprim (R-type ACSSuTTm). To investigate their international spread, we studied the presence of *S. enterica* serovar Enteritidis isolates carrying pUO-SeVR1–like plasmids in the United Kingdom.

## The Study

We screened 31,615 *S. enterica* serovar Enteritidis isolates that had been collected from clinical specimens during 2005–2010 and deposited in the culture collection of the Health Protection Agency *Salmonella* Reference Unit. We screened the isolates for R-type ACSSuTTm. A total of 14 serovar Enteritidis isolates showing this resistance phenotype were detected and subsequently examined for the presence of integron-located *dfrA7*. Of the 14 isolates, 11 were positive and their plasmid content was analyzed by S1 pulsed-field gel electrophoresis (PFGE) (*2*) and by the Kado and Liu methods (*7*); we used serovar Enteritidis strains NRL-Salm-PT4 and CNM4839/03 as controls for pSEV– and pUO-SeVR1–carrying isolates, respectively. The 11 isolates harbored 1 plasmid of variable size (60–95 kb); among these, 9 isolates hybridized with *dfrA7*-specific and *spvC*-specific probes (with plasmids of 85–95 kb) (Figure). These 9 isolates contained a VR-hybrid plasmid similar to pUO-SeVR1 and were selected for further analyses (Table 1, Table 2). The remaining 2 isolates carried the normal pSEV plasmid (60 kb), in which *spvC* hybridized; *dfrA7* was chromosomally located.

**Figure:**
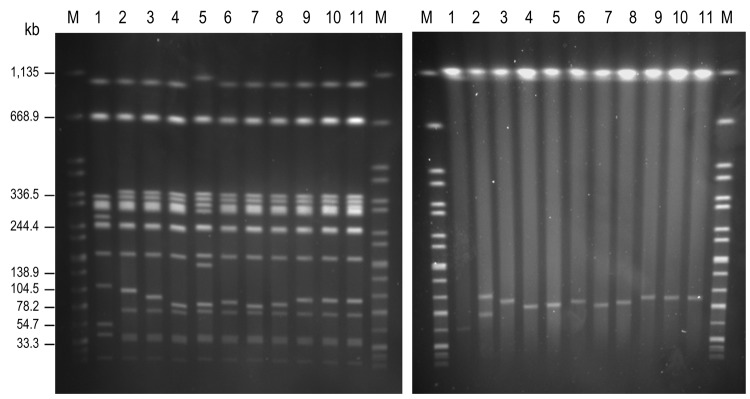
Genomic macrorestriction of *Salmonella* *enterica* serovar Enteritidis isolates: pulsed-field gel electrophoresis profiles for *Xba*I (left panel) and S1 (right panel). Lane M, *Xba*I-digested DNA of *S. enterica* serovar Braenderup H9812, used as size standard; lane 1, NRL-Salm-PT4; lane 2, CNM4839/03; lane 3, H051860415; lane 4, H070360201; lane 5, H070420137; lane 6, H073180204; lane 7, H091340084; lane 8, H091800482; lane 9, H095100307; lane 10, H100240198; lane 11, H101700366. The strain NRL-Salm-PT4 was used as control for the most commonly found *Xba*I-profile in *S*. *enterica* serovar Enteritidis.

**Table 1:** Epidemiologic information for multidrug-resistant *Salmonella* *enterica* serovar Enteritidis isolates, 2005–2010, UK*

Isolate no.	Date of isolation	Source	Recent travel history	African patient name	Patient age, y
CNM4839/03†	2003	Feces	Unknown	No	3
H051860415	2005 Apr 19	Blood	Nigeria	No	38
H070360201‡	2007 Jan 14	Blood	Unknown	Yes	35
H070420137‡	2007 Jan 15	Feces	Unknown	Yes	35
H073180204	2007 Jul 31	Blood	Unknown	Yes	34
H091340084	2009 Mar 15	Feces	Uganda	No	59
H091800482	2009 Apr 17	Blood	Unknown	Yes	30
H095100307§	2009 Dec 7	Blood	Unknown	Yes	68
H100240198§	2010 Jan 9	Blood	Unknown	Yes	68
H101700366§	2010 Apr 22	Blood	Unknown	Yes	68

*All isolates contained a virulence-resistance hybrid plasmid similar to pUO-SeVR1. †Control isolate from Spain. ‡Recovered from the same patient. §Recovered from the same patient.

**Table 2:** Characteristics of *Salmonella* *enterica* serovar Enteritidis isolates harboring pUO-SeVR1-like plasmids, 2005–2010, UK*

Isolate no.	Phage type	Resistance phenotype/ genotype	Class 1 integron†	pSEV genes‡	MLVA	MLST	VR plasmid, kb
CNM4839/03	PT 14b	AMP, CHL, STR, SUL, TET, TMP/*bla*_TEM-1,_ *catA2, strA-strB, sul1, sul2, tet*(A), *dfrA7*	700 bp/*dfrA7*	*spvA, spvB, spvC, spvR, rsk, rck, mig-5,* *srgB, srgC, pefA, pefB, pefC, pefD*	2-12-9-4-4-3-NA-8-8	ST11	100
H051860415	PT 42	AMP, CHL, STR, SUL, TET, TMP/*bla*_TEM-1,_ *catA2, strA-strB, sul1, sul2, tet*(A), *dfrA7*	700 bp/*dfrA7*	*spvA, spvB, spvC, spvR, rsk, rck, mig-5, srgB, srgC, pefA, pefB, pefC, pefD*	2-13-9-4-4-3-NA-8-8	ST1479	95
H070360201	PT 42	AMP, CHL, STR, SUL, TET, TMP/*bla*_TEM-1,_ *catA2, strA-strB, sul1, sul2, tet*(A), *dfrA7*	700 bp/*dfrA7*	*spvA, spvB, spvC, spvR, rsk, rck, mig-5, ΔsrgA, srgB, srgC, pefA, pefB*	2-15-9-4-4-3-NA-8-8	ST1479	88
H070420137	PT 42	AMP, CHL, STR, SUL, TET, TMP/*bla*_TEM-1,_ *catA2, strA-strB, sul1, sul2, tet*(A), *dfrA7*	700 bp/*dfrA7*	*spvA, spvB, spvC, spvR, rsk, rck, mig-5, ΔsrgA, srgB, srgC, pefA, pefB*	2-15-9-4-4-3-NA-8-8	ND	88
H073180204	PT 42	AMP, CHL, STR, SUL, TET, TMP/*bla*_TEM-1,_ *catA2, strA-strB, sul1, sul2, tet*(A), *dfrA7*	700 bp/*dfrA7*	*spvA, spvB, spvC, spvR, rsk, rck, mig-5, srgB, srgC, pefA, pefB, pefC, pefD*	2-13-9-4-4-3-NA-8-8	ND	92
H091340084	PT 42	AMP, CHL, STR, SUL, TET, TMP/*bla*_TEM-1,_ *catA2, strA-strB, sul1, sul2, tet*(A), *dfrA7*	700 bp/*dfrA7*	*spvA, spvB, spvC, spvR, rsk, rck, mig-5, ΔsrgA, srgB, srgC, pefA, pefB, pefC, pefD*	2-9-9-4-4-3-NA-8-8	ST11	90
H091800482	PT 42	AMP, CHL, STR, SUL, TET, TMP/*bla*_TEM-1,_ *catA2, strA-strB, sul1, sul2, tet*(A), *dfrA7*	700 bp/*dfrA7*	*spvA, spvB, spvC, spvR, rsk, rck, mig-5, srgB, srgC, pefA, pefB, pefC, pefD*	2-13-9-4-4-3-NA-8-8	ND	92
H095100307	PT 42	AMP, CHL, STR, SUL, TET, TMP/*bla*_TEM-1,_ *catA2, strA-strB, sul1, sul2, tet*(A), *dfrA7*	700 bp/*dfrA7*	*spvA, spvB, spvC, spvR, rsk, rck, mig-5, ΔsrgA, srgB, srgC, pefA, pefB, pefC, pefD*	2-13-9-4-4-3-NA-8-8	ND	95
H100240198	PT 42	AMP, CHL, STR, SUL, TET, TMP/*bla*_TEM-1,_ *catA2, strA-strB, sul1, sul2, tet*(A), *dfrA7*	700 bp/d*frA7*	*spvA, spvB, spvC, spvR, rsk, rck, mig-5, ΔsrgA, srgB, srgC, pefA, pefB, pefC, pefD*	2-13-9-4-4-3-NA-8-8	ND	95
H101700366	PT 42	AMP, CHL, STR, SUL, TET, TMP/*bla*_TEM-1,_ *catA2, strA-strB, sul1, sul2, tet*(A), *dfrA7*	700 bp/*dfrA7*	*spvA, spvB, spvC, spvR, rsk, rck, mig-5, ΔsrgA, srgB, srgC, pefA, pefB, pefC, pefD*	2-13-9-4-4-3-NA-8-8	ND	95

*pSEV, serovar-specific V plasmid of *S.enterica* serovar Enteritidis; MLVA, multilocus variable number tandem repeat analysis; MLST, multilocus sequence typing; VR, virulence-resistance; PT, phage type; NA, no amplification from this locus; AMP, ampicillin; CHL, chloramphenicol; STR streptomycin; SUL, sulfonamides; TET, tetracycline; TMP, trimethoprim; ST, sequence type; ND, not done. †Size of the variable region amplified with the 5′CS and 3′CS primers (*2*). ‡All plasmids were positive for IncFIIA, IncFIB and the *par* locus. Two new primer pairs were devised for detection of *srgA*: srgAB-Fw1/Rv1 (5′-CGCCTTCCGTGTATGTCC/GCGAGTCACTCACCGACAG-3′) and srgAB-Fw2/Rv2 (5′-GTTGCACAGGAGTGGGAGTC/GTCCGGGTTCCATGTCAG-3′). The forward primers anneal at different positions within *srgA*; the reverse primers anneal at different positions within *srgB*.

In the 9 isolates carrying VR-hybrid plasmids, the R-type ACSSuTTm was encoded by the R-genes *bla*_TEM-1,_ *catA2*, *strA-strB*, *sul1*, *sul2*, *tet*(A), and *dfrA7*, which were located on the pUO-SeVR1–like plasmids as determined by Southern blot hybridization. By PCR amplification, using previously described primers and conditions (*5**,**8*) (Table 2), and by Southern blot hybridization (*5*), we tested for the presence of IncFII and IncFIB replicons, *parAB* (partition), *spvRABCD*, *rck* (resistance to complement killing), *mig-5* (macrophage-induced gene), *pefABCDI* (Pef fimbriae operon), and *srgA* (SdiA-regulated gene; next to *orf7*), all carried by pSEV. The 9 plasmids were positive for the 2 replicons and for all genes screened except *pefC* and *pefD* (absent in H070360201 and H070420137), *pefI*-*orf7* (absent in all), and *srgA* (either absent [H051860415, H073180204, and H091800482] or truncated [in the remaining isolates]) (Table 2).

Isolate subtyping was conducted by phage typing, multilocus variable number tandem repeat analysis (MLVA), multilocus sequence typing (MLST), and *Xba*I-PFGE (*9**,**10*) (http://mlst.ucc.ie/mlst/dbs/Senterica; www.pulsenetinternational.org). The 9 *S. enterica* serovar Enteritidis isolates belong to phage type (PT) 42, in contrast with CNM4839/03, which belongs to PT14b (Table 2). We identified 4 MLVA profiles, which were all single-locus variants of the highly variable locus SENTR5, indicating that the isolates are closely related (Table 2). The isolate from Spain and 3 of the 9 isolates from the United Kingdom, selected as representative of each MLVA profile, were also analyzed by MLST (Table 2). CNM4839/03 and H091340084 were assigned to sequence type (ST) 11, the most commonly found ST in serovar Enteritidis (http://mlst.ucc.ie/mlst/). The remaining 2 isolates could be ascribed to ST1479, the first examples of this single-locus variant of ST11 in the MLST database (Table 2). According to *Xba*I-PFGE, the control strain NRL-Salm-PT4 showed a clearly distinct profile in comparison with CNM4839/03 and the 9 isolates containing pUO-SeVR1–like plasmids, which generated 6 closely related patterns (Figure). Most isolates differed by 1 band of variable size (85–95 kb), which corresponded to the *Xba*I-linearized VR-hybrid plasmids. As an exception, isolate H070420137 showed additional differences in chromosomal bands of ≈150 and ≈300 kb. Because isolates H070420137 and H070360201 came from the same patient (Table 1) and shared the same V and R genotypes and other typing markers, 1 isolate could have evolved from the other; however, co-infection of the patient with 2 closely related strains cannot be ruled out. In addition, considering the typing results of H095100307, H100240198, and H101700366, the identical size of their plasmids, and the fact that they were isolated from the same patient (Table 1), these 3 isolates can be considered the same strain.

All except 2 of the UK isolates carrying pUO-SeVR1–like plasmids were recovered from the blood of patients who had recently returned from an African country or who had an African name (Table 1). Supporting the possible African origin, similar *Xba*I-PFGE profiles and MDR phenotypes (ampicillin, trimethoprim, sulfonamides, and tetracycline) have been identified in clinical *S. enterica* serovar Enteritidis isolates involved in outbreaks and community infections in different African countries. These isolates caused bacteremia, meningitis, diarrhea (*11**–**13*), and high case-fatality rates; they affected mainly children, whereas most clinical isolates analyzed in our study were obtained from adults with bacteremia (Table 1). The detection of the 700-bp/*dfrA7* integron in *S. enterica* serovar Enteritidis isolates from Africa (*14*) also supports an African origin of the MDR serovar Enteritidis isolates harboring pUO-SeVR1–like plasmids. Of note, resistance derivatives of pSLT, the V-plasmid specific to *S*. Typhimurium, have been found in the epidemic ST313 clone of this serovar, which has been considered a major cause of invasive disease in sub-Saharan Africa (*15*).

## Conclusions

Closely related MDR *S. enterica* serovar Enteritidis isolates carrying pUO-SeVR1–like plasmids were recovered in the United Kingdom. Most were isolated from the blood of patients linked to Africa, and they showed common features with serovar Enteritidis isolates involved in outbreaks on that continent. The possibility that this potentially invasive clone of *S. enterica* serovar Enteritidis can be spread through human travel, together with the detection of VR-plasmids in the serovar most frequently associated with human infections, is of public health concern and requires surveillance.
